# Defining the gap: a systematic review of the difference in rates of diabetes-related foot complications in Aboriginal and Torres Strait Islander Australians and non-Indigenous Australians

**DOI:** 10.1186/s13047-017-0230-5

**Published:** 2017-11-07

**Authors:** Matthew West, Vivienne Chuter, Shannon Munteanu, Fiona Hawke

**Affiliations:** 1 Discipline of Podiatry, Faculty of Health and Medicine, University of Newcastle, Box 127, Ourimbah, PO 2258 Australia; 2 Discipline of Podiatry, School of Allied Health, College of Science, Health and Engineering, La Trobe University, Melbourne, Australia; 3 La Trobe Sport and Exercise Medicine Research Centre, College of Science, Health and Engineering, La Trobe University, Melbourne, Australia

**Keywords:** Diabetes, Aboriginal and Torres Strait islander health, Foot, Amputation, Ulceration

## Abstract

**Background:**

The Aboriginal and Torres Strait Islander community has an increased risk of developing chronic illnesses including diabetes. Among people with diabetes, foot complications are common and make a significant contribution to the morbidity and mortality associated with this disease. The aim of this review was to systematically evaluate the literature comparing the rates of diabetes related foot complications in Aboriginal and Torres Strait Islander Australians to non-Indigenous Australians.

**Methods:**

MEDLINE, EMBASE, The Cochrane Library; PUBMED and CINAHL were searched from inception until August 2016. Inclusion criteria were: published cross-sectional or longitudinal studies reporting the prevalence of diabetes related foot complications in both a cohort of Aboriginal and Torres Strait Islander Australians and a cohort of one other Australian population of any age with diabetes. Risk of bias was assessed using the STROBE tool.

**Results:**

Eleven studies including a total of 157,892 participants were included. Studies were set in Queensland, the Northern Territory and Western Australia, primarily in rural and remote areas. Aboriginal and Torres Strait Islander Australians experienced substantially more diabetes related foot complications with the mean age up to 14 years younger than non-Indigenous Australians. Aboriginality was associated with increased risk of peripheral neuropathy, foot ulceration and amputation. In several studies, Aboriginal and Torres Strait Islander Australians accounted for the vast majority of diabetes related foot complications (up to 91%) while comprising only a small proportion of the regional population. Reporting quality as assessed with the STROBE tool showed underreporting of: methods, sample description and potential sources of bias. There are no data available for some Australian states and for specific types of diabetes related foot complications.

**Conclusions:**

Aboriginal and Torres Strait Islander Australians have a 3–6 fold increased likelihood of experiencing a diabetes related foot complication compared to non-Indigenous Australians. Evidence-based, culturally appropriate screening and intervention programs and improved access to effective health care services are required to prevent a widening of the gap in diabetes related foot complications between Aboriginal and Torres Strait Islander and non-Indigenous Australians.

## Background

The Aboriginal and Torres Strait Islander community has increased risk of developing chronic illness including diabetes [1]. Among people with diabetes, foot complications are common and make a significant contribution to the morbidity and mortality associated with the disease [2]. In 2008, the age-standardised rate of diabetes was nearly three and four times greater among Aboriginal and Torres Strait Islander men and women respectively when compared to non- Indigenous Australians [3]. This same report found diabetes accounted for 16% of all hospitalisations within the Aboriginal and Torres Strait Islander community, was the primary cause of hospitalisation in 6% of all hospitalisations and an associated diagnosis in 11% of all hospitalisations [3]. Diabetic complications have also been found to make up the majority [67%] of preventable hospitalisations for chronic conditions for Aboriginal and Torres Strait Islander Australians [4], significantly contributing to the seven-fold increase risk of diabetes-related mortality in this population [5].

For Aboriginal and Torres Strait Islander Australians, development of complications secondary to diabetes commonly precedes the diagnosis of diabetes itself [6]. Such complications include retinopathy, nephropathy and neuropathy [2] and indicate a patient has had prolonged periods of hyperglycaemia [7]. The chronic nature of these conditions renders them difficult to treat. Recommended sustained lifestyle modifications and intensive multidisciplinary team action have varied effectiveness [8], especially when compared to the benefits of prevention through early effective management and education [9]. The challenge of such management is further exacerbated by higher prevalence of known unhealthy lifestyle behaviours such as smoking, and high rates of obesity in the Aboriginal and Torres Strait Islander community which are associated with poorer treatment outcomes [5]. In the foot, the secondary complications of diabetes often culminate in ulceration, chronic wounds, infection and amputation [2].

Considerably higher rates of diabetes among Aboriginal and Torres Strait Islander Australians suggest the existing trend of higher diabetes associated hospitalisation among this population compared to their aged match non-Indigenous peers will continue [3]. Although estimated to be higher than in the general population, little is known about rates of diabetes related foot complications in Aboriginal and Torres Strait Islander Australians, making development and implementation of targeted, effective prevention and management strategies challenging [4]. Therefore, the aim of this review was to systematically evaluate the literature reporting rates of diabetes related foot complications for Aboriginal and Torres Strait Islander Australians compared to non-Indigenous Australians.

## Methods

**Fig. 1:**
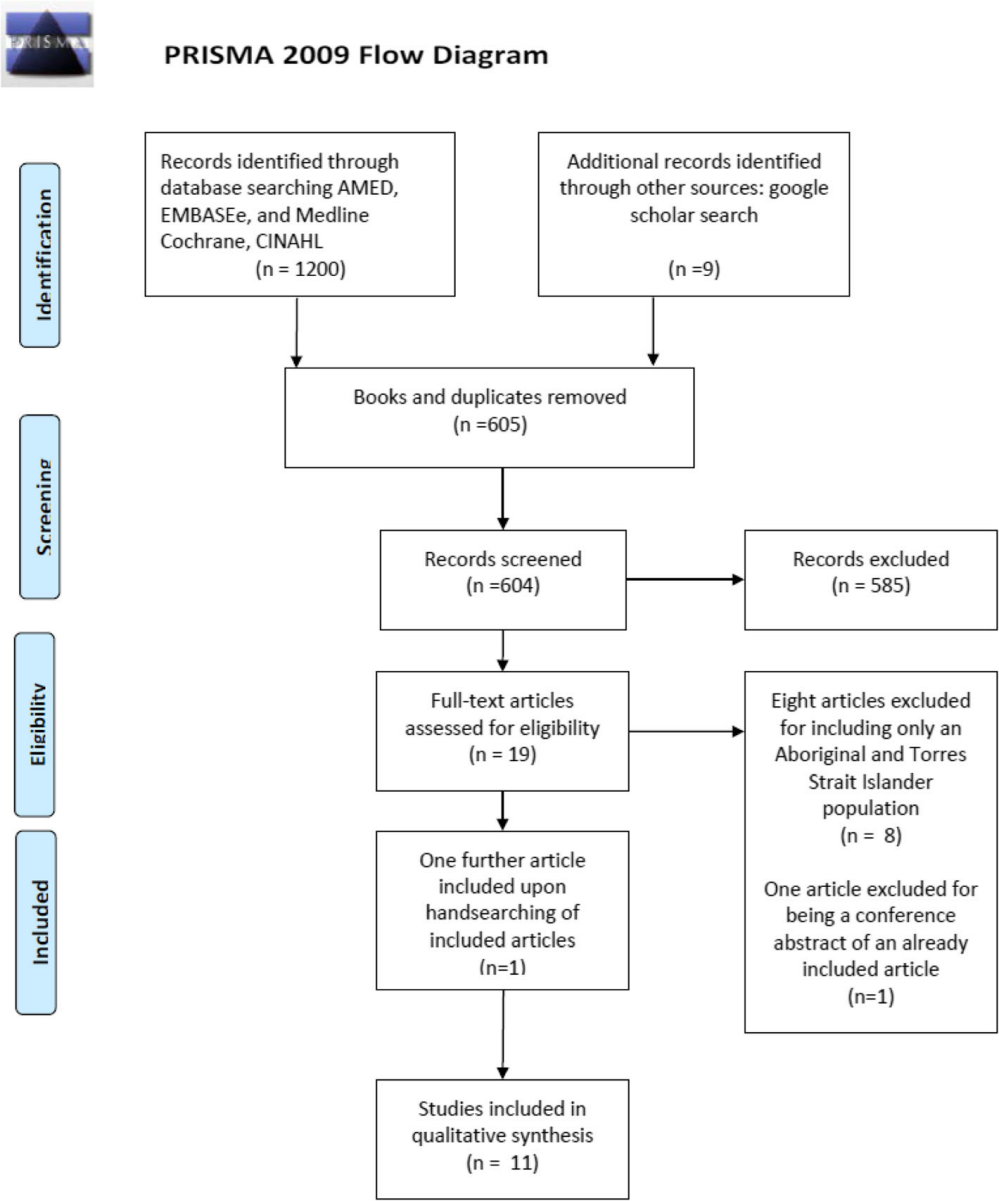
PRISMA flow diagram

This systematic review was developed and reported according to the guidelines provided by the Preferred Reporting of Systematic Reviews and Meta-Analysis (PRISMA) as seen in Fig. 1.

### Data sources

An electronic database search was conducted in August 2016 of MEDLINE (January 1966 to present) EMBASE (January 1980 to present); Cochrane Central Register of Controlled Trials (CENTRAL) (The Cochrane Library, latest issue); PUBMED (January 1966 to present); CINAHL (from 1982). The keyword terms used in different combinations were: Aborig*, Indigenous, Australia*, Lower limb, Foot, Leg, Ankle, Ulcer, Neuro*, Amputat*, Diabet*, Vascular, Peripheral arterial disease, Ischaemi*. No language, publication date or publication status restrictions were used but only original published literature were eligible for inclusion. The MEDLINE search string is provided in Table 1 as an example. The following search strategies were used to supplement electronic searches: checking of reference lists of included studies, relevant clinical guidelines and review articles; citation tracking for included studies; and contacting prominent authors in the field.

**Table 1:** Example of search string - Ovid SP Medline

1.	Aborig*
2.	Indigenous
3.	1 or 2
4.	Australia*
5.	3 and 4
6.	Lower limb
7.	Foot
8.	Leg
9.	Ankle
10.	Ulcer
11.	Neuro*
12.	Amputat*
13.	Vascular
14.	Peripheral arterial disease
15.	Ischaemi*
16.	6–15/or
17.	5 and 16

The asterisk (*) truncation symbol was used as a substitute for any string of zero or more characters in a search term. For example, the search diabet* would include diabetes, diabetic etc.

Published studies providing cross-sectional or longitudinal data on the prevalence of diabetes related foot complications in both a cohort of Aboriginal and Torres Strait Islander Australians and a cohort of one other Australian population of any age with diabetes were eligible for this review. Studies reporting prevalence of foot complications in Aboriginal and Torres Strait Islander Australians only were excluded as comparative regional data for non-Indigenous Australians is required to determine the extent of the disparity in diabetes-related complications in Aboriginal and Torres Strait Islander Australians. Included studies were required to report foot complications associated with diabetes, including lower limb amputation of any level, cellulitis, Charcot neuroarthropathy, intermittent claudication, ischaemia, necrosis, sensory, autonomic and/or motor neuropathy, peripheral arterial disease, ulceration and/or wound infection. One reviewer conducted the electronic searches (MW). Titles and abstracts were independently assessed by two reviewers (MW and FH). Disagreements were resolved by discussion. A third reviewer (VC) was to arbitrate disagreements but this was not required.

### Data extraction

MW extracted study data using standardised, pilot-tested data extraction forms, which were checked for transcription errors and completeness by FH. Data were extracted for eligibility criteria, mean age and age range of the cohort, gender, ethnicity, geographic region (state and urban/remote setting), study types (including population based and hospital audits), method of data collection for complications (i.e. measured, hospital records, self-report), type of diabetes, and type of complication.

### Assessment of study quality

Completeness of reporting was assessed independently by MW and FH using the STROBE checklist. The STROBE tool is a series of questions set out as a check list. See supplementary material. Articles are then scored as reporting or not reporting each item to give an overall view of completeness of reporting [10]. Disagreements were resolved by discussion. No record was kept of disagreements. A third reviewer (VC) was to arbitrate disagreements that were not easily resolved but again, this was not required.

### Analysis

Data are reported as whole numbers and/or percentages for prevalence or incidence, and odds ratios (OR) for magnitude of difference between Aboriginal and Torres Strait Islander and non-Indigenous groups where this was reported. Due to heterogeneity between studies no meta-analysis was performed.

## Results

### Overview of included studies

Electronic searching retrieved 1209 articles. All books (437) and duplicates (168) were excluded leaving 604 articles to be screened (Fig. 1)**.** Of these, 585 were not relevant to the topic and 19 were potentially relevant. All potentially relevant studies were reviewed in full text. Eight articles were then excluded for including only an Aboriginal and Torres Strait Islander cohort [11–18]. One further article [19] was then excluded for being a conference abstract of an already included study [20] which upon contact with the author was confirmed to offer no further relevant data. Ten remaining articles met all inclusion criteria for this review [20–29]. Three of the ten included articles were produced from the one study, the Fremantle Diabetes Study [18, 23, 24]. Hand searching for articles reporting the Fremantle Diabetes Study identified one further study for inclusion [30]. Therefore, 11 articles were included, and four of those reported in the Fremantle Diabetes Study.

### Characteristics of included studies

A summary of study characteristics is included in Table 2. All studies included both an Aboriginal and Torres Strait Islander cohort and one other non-Indigenous cohort. Two studies did not report sample sizes [25, 26]. Of the remaining studies sample sizes ranged from 51 [31] to 120,097 [24]. Regions studied includes Cairns and Townsville in Queensland reported in four studies [20, 27–29], Tennant Creek and Darwin in the Northern Territory [22, 25] and one state-wide study in Western Australia [26] and one study in Fremantle (published as four papers) [21, 23, 24, 32] . Most studies were audits of previously collected data [22, 25, 26–29] with the exception of one study [20] which collected data at the time of occasion of service in hospital inpatients. Audit data included three specific hospital audits [25, 27, 29], one high risk foot clinic audit [28], one audit of a dialysis unit [20] and one audit of amputation data for the state of Western Australia (WA) [26].

**Table 2:** Summary of included studies

Study ID	Study design	Location	n. of ATSI (as % of sample)	Finding
Commons [22]	Prospective single sample review of consecutive inpatients with diabetic foot infections	Darwin, NT	144 (81.4)	Indigenous people had a greater incidence of admission (RR: 5.1; 95%CI: 3.8 to 7.0), were younger (mean difference: 11.1 years; *p* < 0.001), and more likely to undergo major amputations (RR: 4.1; 95%CI: 1.6 to 10.7), and minor amputation (RR: 6.2; 95%CI: 3.5 to 11.1). Non-multiresistant methicillin resistant *S. aureus* was present in more wounds for Indigenous people than non-Indigenous patients (44.7% vs. 20.6%; OR: 3.1; 95%CI: 1.5 to 6.4), whereas *P. aeruginosa* presence was significantly less (15.8% versus 46.0%; OR: 0.22; 95%CI: 0.11 to 0.45). Rate of known peripheral vascular disease was lower among Indigenous people (13.2% vs. 34.9%; *p* = 0.001). Rate of prior amputation among indigenous people was higher (33.3% vs. 19.0%; *p* = 0.043). There was no important difference in prevalence of osteomyelitis between ATSI (36.0%) and non-ATSI (34.9%).
Ewald [25]	Clinical audit of two hospitals in Alice Springs and Tennant Creek	Tennant Creek, NT	Not reported	Indigenous people made up 89% of individuals with foot complications and 91% of separations for diabetic foot but comprised only 38% of the total regional population.
Gilhotra [20]	Clinical audit of Dialysis Centre	Townsville, QLD	113 (51.8)	Indigenous status was independently associated with lower limb amputation (OR: 4.98; 95%CI: 1.3 to 19.23; *p* = 0.02) in people with end-stage renal failure on dialysis.
Norman [26]	Clinical audit of all lower limb amputations in WA	WA, state wide	Not reported	Among people 25 to 49 years of age with diabetes, major amputations were 38 times more likely and minor amputations 27 times more likely in ATSI than non-ATSI. 98% of amputations in Indigenous people were associated with diabetes.
O’Rourke [27]	Clinical audit of 143 diabetes mellitus-related major amputations between 1998 and 2008	Cairns Base Hospital, QLD	74 (51.7)	ATSI people accounted for 51.7% of the 143 major diabetes-related amputations performed yet comprised about 9.6% of the regional population. PAD was diagnosed in 48.6% of ATSI and 11.6% of non-ATSI who underwent amputation. The mean age at the time of amputation was 56.3 years for ATSI, 14 years younger than that for non-ATSI. Pressure ulcers necessitated amputation in 4.1% of ATSI and 4.3% of non-ATSI.
Rodrigues [28]	Clinical audit of people attending the high risk foot clinic	Townsville, QLD	23 (17.8)	In people with diabetic foot ulcers, Indigenous ethnicity was independently associated with lower limb amputation (OR: 3.1; 95%CI: 1.17 to 9.16; *p* = 0.001). The mean age at amputation was similar between ATSI (mean yrs. 62.6; SD 12.5) and non-ATSI (mean yrs. 62.0; SD 11.5).
Steffen [29]	Clinical audit of 51 patients admitted to with diabetic foot complications that required surgical intervention	Cairns Base Hospital, QLD	29 (56.9)	Indigenous people accounted for 57% of audit cases yet comprised 13% of the regional population. Mean age at surgical intervention was 9.5 years younger in ATSI than non-ATSI people (56.5 vs. 66.0).
Davis [30)]	Series of longitudinal observational studies	Fremantle, WA	18 (2.2)	At baseline in people with type 2 diabetes, there were no statistically significant differences between ATSI and non-ATSI in prevalence of neuropathy (41.2% vs. 32.9%; *p* = 0.45); PAD (16.7% vs. 29.5%; *p* = 0.30) or foot ulceration (5.6% vs. 1.2%; *p* = 0.22).
Davis [24]			Baseline = 37 (3.0)	In 1237 people with type 2 diabetes, Aboriginal background was identified as an independent risk factor for neuropathy (OR: 3.7; 95%CI: 1.17–11.70; *p* = 0.03)
Davis [23]			Phase 1 = 19 (2.3) Phase 2 = 106 (11.8)	At baseline in phase 1, there were no statistically significant differences between ATSI and non-ATSI in prevalence of peripheral sensory neuropathy (38.9% vs. 33.6%; *p* = 0.62) or PAD (15.8% vs. 29.7%; *p* = 0.31). At baseline in phase 2, there were statistically significant differences between ATSI and non-ATSI in the prevalence of peripheral sensory neuropathy (48.5% vs. 63.3%; *p* = 0.005) and PAD (30.7% vs. 21.5%; *p* = 0.04).
Baba [21]			120 (4.3)	Aboriginality was independently associated with foot ulcer at baseline in pooled phase samples (OR: 4.8; 95%CI: 1.7–13.7; *p* = 0.004).

*ATSI* Aboriginal and Torres Strait Islander, *RR* Rate ratio, *OR* Odds ratio, *PAD* Peripheral Arterial/Vascular Disease, *T2DM* Type 2 Diabetes Mellitus, *QLD* Queensland, *WA* Western Australia, *NT* Northern Territory

The Fremantle Diabetes Study consisted of a series of cross-sectional studies conducted as two phases between 1993 to 1996 and 2008 to 2011 [21, 23, 24, 30]. Participants in the Fremantle Diabetes study were recruited from centres of health care provision within the same zip code defined region of the city of Fremantle. Centres included but were not limited to: inpatient and outpatient hospital clinics; primary care and specialist physicians; allied health services; and pharmacies [30]. Findings are presented in Table 2.

### Rates of foot complications in reported populations

Thematic analysis of extracted data from included studies relating to specific foot complications including amputation, peripheral neuropathy, peripheral arterial disease (PAD), ulceration and infection is shown in Table 3. Rates of amputation were consistently higher in Aboriginal and Torres Strait Islander cohorts than in non-Indigenous cohorts [20, 22, 26–28]. The greatest difference was reported by Norman et al. [26]. In their clinical audit of all lower limb amputations in WA there was a 38 fold higher rate of major lower limb amputation and a 27 fold higher rate of minor amputation among Aboriginal and Torres Strait Islander Australians 25 to 49 years of age with diabetes [26]. Aboriginal and Torres Strait Islander Australians were also more likely to be admitted to hospital for diabetic foot ulcerations [22] and to require surgical intervention for diabetic foot complications (four times greater for major amputation and six times greater for minor amputations) [29]. Findings of the Fremantle Diabetes Study were inconsistent between study phases, possibly due to different study populations and study timing, and due to the small proportion of Aboriginal and Torres Strait Islander Australians included. Pooling of participants from baseline in both phases found that Aboriginality was independently associated with foot ulcer (OR: 4.8; 95% confidence intervals [CI]: 1.7–13.7; *p* = 0.004) [23].Two studies referred to the population of the geographical region they were examining at the time of publication as a benchmark to establish that Aboriginal and Torres Strait Islander Australians experienced the majority of foot complications while comprising a minority of the regional population [25, 29]. Complications examined in both studies included infection and amputation.

**Table 3:** Thematic summary of included study findings

Reported Theme	Study	Key findings
Amputation	Commons [22]	Indigenous people had a greater incidence of major amputations (RR: 4.1; 95% CI: 1.6 to 10.7), and minor amputation (RR: 6.2; 95% CI: 3.5 to 11.1). Rate of prior amputation among indigenous people was higher (33.3% vs. 19.0%; *p* = 0.043).
	Ewald [25]	Indigenous people made up 89% of individuals with foot complications and 91% of separations for diabetic foot but comprised only 38% of the total regional population.
	Gilhotra [20]	Indigenous status was independently associated with lower limb amputation (OR: 4.98; 95% CI: 1.3 to 19.23; *p* = 0.02) in people with end-stage renal failure on dialysis.
	Norman [26]	Among people 25 to 49 years of age with diabetes, major amputations were 38 times more likely and minor amputations 27 times more likely in ATSI than non-ATSI. 98% of amputations in Indigenous people were associated with diabetes.
	O’Rourke [27]	ATSI people accounted for 51.7% of the 143 major diabetes-related amputations performed yet comprised about 9.6% of the regional population. The mean age at the time of amputation was 56.3 years for ATSI, 14 years younger than that for non-ATSI.
	Rodrigues [28]	Indigenous ethnicity was independently associated with lower limb amputation (OR: 3.1; 95% CI: 1.17 to 9.16; *p* = 0.001). The mean age at amputation was similar between ATSI (mean yrs. 62.6; SD 12.5) and non-ATSI (mean yrs. 62.0; SD 11.5).
	Steffen [29]	Indigenous people accounted for 57% of audit cases yet comprised 13% of the regional population. Mean age at surgical intervention was 9.5 years younger in ATSI than non-ATSI people (56.5 vs. 66.0).
PAD	Commons [22]	Rate of known peripheral vascular disease was lower among Indigenous people (13.2% vs. 34.9%; *p* = 0.001)
	O’Rourke [27]	PAD was diagnosed in 48.6% of ATSI and 11.6% of non-ATSI who underwent amputation.
	Davis [23]	At baseline in people with type 2 diabetes, there were no statistically significant differences between ATSI and non-ATSI in prevalence of); PAD (16.7% vs. 29.5%; *p* = 0.30) At baseline in phase 1, there were no statistically significant differences between ATSI and non-ATSI in prevalence of PAD (15.8% vs. 29.7%; *p* = 0.31). At baseline in phase 2, there were statistically significant differences between ATSI and non-ATSI in the prevalence of PAD (30.7% vs. 21.5%; *p* = 0.04).
Peripheral Neuropathy	Davis [30]	At baseline in people with type 2 diabetes, there were no statistically significant differences between ATSI and non-ATSI in prevalence of neuropathy (41.2% vs. 32.9%; *p* = 0.45);
	Davis [24]	In 1237 people with type 2 diabetes, Aboriginal background was identified as an independent risk factor for neuropathy (OR: 3.7; 95% CI: 1.17–11.70; *p* = 0.03
	Davis [23]	At baseline in phase 1, there were no statistically significant differences between ATSI and non-ATSI in prevalence of peripheral sensory neuropathy (38.9% vs. 33.6%; *p* = 0.62) At baseline in phase 2, there were statistically significant differences between ATSI and non-ATSI in the prevalence of peripheral sensory neuropathy (48.5% vs. 63.3%; *p* = 0.005
Ulceration	O’Rourke [27]	Pressure ulcers necessitated amputation in 4.1% of ATSI and 4.3% of non-ATSI.
	Rodrigues [28]	In people with diabetic foot ulcers, Indigenous ethnicity was independently associated with lower limb amputation (OR: 3.1; 95%CI: 1.17 to 9.16; *p* = 0.001).
	Baba [21]	Aboriginality was independently associated with foot ulcer at baseline in pooled phase samples (OR: 4.8; 95% CI: 1.7–13.7; *p* = 0.004).
Infection	Commons [22]	Non-multi-resistant methicillin resistant S. aureus was present in more wounds for Indigenous people than non-Indigenous patients (44.7% vs. 20.6%; OR: 3.1; 95%CI: 1.5 to 6.4), whereas P. aeruginosa presence was significantly less (15.8% versus 46.0%; OR: 0.22; 95% CI: 0.11 to 0.45).

In studies that reported rates of foot complications alongside regional populations, Aboriginal and Torres Strait Islander Australians experienced the majority of foot complications while comprising a minority of the regional population [25, 29].

### Quality appraisal

The STROBE tool used to summarise completeness of reporting is available as Online Supplement 1. Completeness of reporting varied across studies. All included studies reported study setting and location, eligibility criteria and number of individual participants. Despite this, in most studies demographic information specific to the sample of included Aboriginal and Torres Strait Islander Australians was not provided. This limits generalisability of the findings. Papers reporting the Fremantle Diabetes Study [21, 23, 24, 30] were generally more completely reported. Among other papers, quality of reporting tended to be better in recent publications. Underreporting of methods and sample description may in some cases be due to the brevity of the publications. This is particularly true of Norman et al., which, despite containing important data, was published as a letter to the editor [26].

## Discussion

Based on the limited data that are currently available, our review has demonstrated higher rates of both diabetes related foot ulcer and lower limb amputation in Aboriginal and Torres Strait Islander Australians compared to the non-Indigenous population. This finding was consistent across all geographical areas included in this review (i.e. regional and rural areas in Queensland and the Northern Territory as well as Western Australia). Aboriginality was shown to be an independent risk factor for diabetes related foot complications resulting in a three to fivefold increased likelihood [20, 28], and sixfold increased relative risk [22] of a lower limb amputation and fivefold increased likelihood of foot ulcer [21] as detailed in Table 3. Similarly, Aboriginal and Torres Strait Islander Australians had a fourfold increased likelihood of peripheral neuropathy compared to a non-Indigenous population [24].

Consistently, Aboriginal and Torres Strait Islander Australians were shown to experience higher rates of amputation than their non-Indigenous counterparts, despite making up a smaller proportion of the populations. O’Rourke et al. [27] reported that in a region where Aboriginal and Torres Strait Islander Australians comprised less than 10% of the regional population, they accounted for more than half of all amputations. Norman et al. [26] when examining trends in amputation for arterial disease or diabetes related separations, found that of individuals 25 to 49 years of age with diabetes, Aboriginal and Torres Strait Islander people were 38 times more likely than non-Indigenous Australians to undergo a major amputation and 27 times more likely to undergo a minor amputation. The increased risk of lower limb amputation among Aboriginal and Torres Strait Islander people was also found among people with end-stage renal failure on dialysis [20].

In the Fremantle Diabetes study, Aboriginality was independently associated with neuropathy [24] and foot ulceration [21]. This is consistent with findings of several other studies in the review, including an independent association between Aboriginality and increased risk of progression of foot ulcer to amputation by O’Rouke et al. [27], and increased risk of hospitalisation with diabetic foot infection for Aboriginal and Torres Strait Islander people [22]. Of people admitted to hospital with diabetic foot infections, Aboriginal and Torres Strait Islander people were reported to be younger, and at greater risk of minor and major amputations than non-Indigenous Australians, despite no significant difference in peripheral vascular disease or osteomyelitis [22].

Similar findings were reported for Indigenous populations in New Zealand, Canada and United States of America. In Canada, for example, Indigenous Canadians experience diabetes and its complications at a higher prevalence and at a younger age than non-Indigenous Canadians [33]. Compared to non-Indigenous Canadians, Indigenous Canadians are four times more likely to have diabetes and 16 times more likely to experience diabetes related foot complications [33]. In New Zealand, Maori people are more likely than people of other ethnicities to have lower limb amputation secondary to type 2 diabetes, even after adjusting for demographic variables [32]. Differences in rates of diabetes-related foot complications between Indigenous and non-Indigenous populations in high income countries discussed above may be a result of historical (and in some parts, continuing) social exclusion and discrimination, reduced health care literacy and access, modifiable lifestyle factors, and lack of culturally appropriate screening and early intervention programs.

A commonly reported finding was that amputation occurs considerably earlier among Aboriginal and Torres Strait Islander populations than their non-Indigenous peers. For example, Norman et al. [26] reported Aboriginal and Torres Strait Islander people aged 25 to 49 years had a 27 times higher risk of incurring a minor diabetes related lower limb amputation. One possible explanation may be that as well as experiencing a higher rate of type two diabetes compared to their age match non-Indigenous peers [3], Aboriginal and Torres Strait Islander populations from a young age (youth aged 12–15) were found to be up to 57% more likely to have poor diet, increased BMI and be smokers [3]. The increased prevalence of such lifestyle risk factors are known to be associated with the increased prevalence of diabetes related lower limb complications (including hospitalisations, surgery and amputation).

Despite evidence that the prevalence of diabetes is higher, growing faster and causing more hospitalisation within the Aboriginal and Torres Strait Islander community [3, 4] there is little published evidence on specific interventions programs for this community. The evidence that is available reports health practitioner knowledge about diabetes related lower limb complications in rural and remote areas is low, and the ability to determine early stage risk of lower limb complication among broad populations with diabetes in these regions is poor [34]. This is of significant relevance to the Aboriginal and Torres Strait Islander population as our review indicates that communities in rural and remote areas were found to experience the greatest rates of amputation [25, 26, 27, 29].

Current evidence supports the use of culturally appropriate intervention to increase short to medium term knowledge of diabetes among ethnic minorities [35]. There are a number of examples of new services that have improved access to diabetes care service and patient outcomes in Aboriginal and Torres Strait Islander communities in Australia. These share common characteristics, including community consultation in the development, implementation and ongoing management of the service; involvement of Aboriginal Health Workers, and a focus on self-management and patient participation in health through improved health literacy [36–38]. While there are also several foot care programs that have been developed to target prevention of foot complications in Aboriginal and Torres Strait Islander populations, there has been limited published evaluation of the success of these in reducing rates of foot complications [31, 39]. Our review findings highlight an urgent need for culturally appropriate foot care intervention programs to be comprehensively evaluated, and for effective programs to be widely implemented to reduce rates of diabetes related foot complications and associated morbidity and mortality in Aboriginal and Torres Strait Islander Australians. As called for by the *Close the Gap Progress and Priorities Report* of 2015, a much greater focus on access to appropriate primary health care services (including early diagnosis, intervention and education) is required to improve health and life expectancy for Aboriginal and Torres Strait Islander Australians [5].

### Limitations

This review aimed to report data on the prevalence of diabetes related foot complications in Aboriginal and Torres Strait Islander Australians compared to that of non-Indigenous Australians. While we performed an exhaustive search for relevant literature, other forms of publications (e.g. government reports) were not included in this review. Furthermore, the purpose of this review was to compare rates of diabetes related foot complications in Aboriginal and Torres Strait Islander Australians and non-Indigenous Australians, therefore while there are some data relating to Aboriginal and Torres Strait Islander people only, these were not included as without comparable regional data these studies would not add to the findings of this paper. This review has highlighted the limited data that are currently available comparing diabetes related foot complications in Aboriginal and Torres Strait Islander Australians and non-Indigenous populations in Australia. Available data mainly focuses on amputation rates and there is little information relating to other diabetes related foot complications e.g. Charcot neuroarthropathy and peripheral arterial disease. As the data from the retrieved studies were specific to several geographical regions in Australia (i.e. Western Australia, Northern Territory and Queensland) the results of this review are not necessarily generalisable nationally. Nevertheless, these findings do highlight consistently high rates of diabetes related foot complications in Aboriginal and Torres Strait Islander Peoples across several geographic regions and the need for related research at a national level to better inform future health care practice.

The quality of research and reporting in studies included in the systematic review varied. Longitudinal studies tended to collect data for more variables and were therefore better able to identify factors associated with diabetes related foot complications. For some audits, data summaries were limited to descriptive statistics. Future research should include detailed sample descriptions, careful description of how diabetes related foot complications are diagnosed and statistical measures of spread. Importantly, for audits comparing Aboriginal and Torres Strait Islander and non-Indigenous populations, researchers should state the proportion of people within the health system of unknown Aboriginal and Torres Strait Islander /non-Indigenous status.

## Conclusion

Aboriginal and Torres Strait Islander Australians experience substantially more diabetes related foot complications than non-Indigenous Australians. This disparity is seen clearly in rates of foot ulceration and amputation. In the limited available comparative data, we found Aboriginal and Torres Strait Islander Australians had between a 3–6 fold increased likelihood of both foot ulcer and minor or major amputation and that these occurred at a younger age. There are a lack of nationwide data relating to diabetes related foot complications in Aboriginal and Torres Strait Islander Australians, and most data focuses on amputation rates with little information available regarding specific types of diabetic foot complications. Greater knowledge of the breadth and depth of this critical problem is required to fully inform implementation of effective evidence-based culturally appropriate screening and intervention programs.

## Abbreviations

ATSI: Aboriginal and Torres Strait Islander
NT: Northern Territory
OR: Odds ratio
PAD: Peripheral Arterial/vascular Disease
QLD: Queensland
RR: Rate ratio
T2DM: Type 2 Diabetes Mellitus
WA: Western Australia
